# A Novel Approach for the Detection and Severity Grading of Chronic Obstructive Pulmonary Disease Based on Transformed Volumetric Capnography

**DOI:** 10.3390/bioengineering11060530

**Published:** 2024-05-23

**Authors:** Xiuying Mou, Peng Wang, Jie Sun, Xianxiang Chen, Lidong Du, Qingyuan Zhan, Jingen Xia, Ting Yang, Zhen Fang

**Affiliations:** 1 Aerospace Information Research Institute, Chinese Academy of Sciences, Beijing 100094, China; mouxiuying18@mails.ucas.ac.cn (X.M.); wangpeng01@aircas.ac.cn (P.W.); sunjie2602215@163.com (J.S.); chenxx@aircas.ac.cn (X.C.); lddu@mail.ie.ac.cn (L.D.); 2 School of Electronic, Electrical and Communication Engineering, University of Chinese Academy of Sciences, Beijing 100049, China; 3 Department of Pulmonary and Critical Care Medicine, Center of Respiratory Medicine, China–Japan Friendship Hospital, Beijing 100029, China; zhanqy@163.com (Q.Z.); xiajingen_00632@163.com (J.X.); 4 Research Unit of Personalized Management of Chronic Respiratory Disease, Chinese Academy of Medical Sciences, Beijing 100190, China

**Keywords:** volumetric capnography, COPD, deep learning

## Abstract

Chronic Obstructive Pulmonary Disease (COPD), as the third leading cause of death worldwide, is a major global health issue. The early detection and grading of COPD are pivotal for effective treatment. Traditional spirometry tests, requiring considerable physical effort and strict adherence to quality standards, pose challenges in COPD diagnosis. Volumetric capnography (VCap), which can be performed during natural breathing without requiring additional compliance, presents a promising alternative tool. In this study, the dataset comprised 279 subjects with normal pulmonary function and 148 patients diagnosed with COPD. We introduced a novel quantitative analysis method for VCap. Volumetric capnograms were converted into two-dimensional grayscale images through the application of Gramian Angular Field (GAF) transformation. Subsequently, a multi-scale convolutional neural network, CapnoNet, was conducted to extract features and facilitate classification. To improve CapnoNet’s performance, two data augmentation techniques were implemented. The proposed model exhibited a detection accuracy for COPD of 95.83%, with precision, recall, and F1 measures of 95.21%, 95.70%, and 95.45%, respectively. In the task of grading the severity of COPD, the model attained an accuracy of 96.36%, complemented by precision, recall, and F1 scores of 88.49%, 89.99%, and 89.15%, respectively. This work provides a new perspective for the quantitative analysis of volumetric capnography and demonstrates the strong performance of the proposed CapnoNet in the diagnosis and grading of COPD. It offers direction and an effective solution for the clinical application of capnography.

## 1. Introduction

Chronic Obstructive Pulmonary Disease (COPD) is a respiratory disease characterized by limited airflow [1]. As one of the major chronic diseases in the world, COPD has brought a significant healthcare burden, particularly in developing countries [2]. According to a World Health Organization (WHO) survey, COPD led to 3.3 million deaths in 2019 [3]. Since it is incurable, early diagnosis and treatment are essential for patients.

Spirometry is considered the gold standard for the initial assessment of COPD, as endorsed by the Global Initiative for Chronic Obstructive Lung Disease (GOLD, 2023) [4]. The diagnosis of COPD is confirmed when the ratio of forced expiratory volume in one second (FEV1) to forced vital capacity (FVC) after the administration of a bronchodilator is less than 0.7, indicating airflow limitation. Furthermore, the FEV1 percentage of predicted normal values (FEV1% predicted) is utilized to grade the severity of airflow limitation in COPD patients. However, forced spirometry tests have stringent performance and interpretation criteria, requiring patients to forcefully and rapidly expel all air from their lungs within a brief timeframe [5]. It is difficult for individuals with impaired respiratory function to perform forced spirometry adequately, especially for the elderly, young children, and patients with severe respiratory distress or neurological conditions that impede their ability to understand or perform the test.

Given the limitations of spirometry, volumetric capnography (VCap) has emerged as a promising alternative. Owing to its non-invasiveness and minimal patient cooperation requirement, VCap has garnered significant interest among researchers. Volumetric capnography, which measures the CO_2_ concentration throughout the entire exhalation, offers detailed insights into both gas exchange and lung ventilation dynamics across different lung volumes [6]. Unlike spirometry, volumetric capnography can be effortlessly performed during natural breathing, making it particularly suitable for vulnerable populations such as infants, the elderly, and those with significant pulmonary impairment. The interpretation of volumetric capnography waveforms currently depends heavily on visual analysis by healthcare professionals, leading to potential misinterpretations. The integration of advanced computational techniques could standardize and enhance the accuracy of waveform analysis.

Many studies have used morphological and statistical features—such as variations in slope and angle, peak values, and averages—to achieve VCap quantitative analyses that are indicative of underlying pulmonary function and pathology [7,8,9]. Distinct waveform patterns observed in volumetric capnography have been instrumental in differentiating between obstructive and restrictive lung diseases. Pertzov et al. used the waveform features of capnography to establish a regression model of FEV1 to assess lung function in patients with COPD and asthma [10]. These researches emphasize extracting predefined statistical parameters from the waveforms and finding their correlations with FEV1 and FVC. Studies have shown that waveform characteristics of volumetric capnography provide significant evidence for discriminating diseases like COPD, asthma, and heart failure [10,11,12]. Typically, capnograms are considered as one-dimensional waveform data. Considering capnograms as one-dimensional waveform data facilitates their integration into machine learning models by allowing for the systematic extraction of temporal features, which is crucial for diagnosing and classifying respiratory conditions. Some researchers have extracted key features for use as inputs to machine learning classifiers to achieve disease diagnosis and classification [13,14]. Mieloszyk, R., et al. have attempted to construct end-to-end models, inputting raw volumetric capnography data directly into the network to differentiate COPD–normal and COPD–CHF [15]. Quantitative analysis methods of capnography based on artificial intelligence technologies offer crucial insights for the diagnosis and assessment of respiratory diseases. The advancement of artificial intelligence, especially through deep learning techniques, has revolutionized the field of medical diagnostics. With the advancement of artificial intelligence, particularly deep learning, substantial potential has been demonstrated in disease diagnosis, such as cardiovascular diseases [16,17,18,19] and cancer diagnosis [20,21,22]. Deep learning, particularly Convolutional Neural Networks (CNN), stands out for its proficiency in image-based analysis, adept at deciphering complex patterns and extracting meaningful insights from high-dimensional data. 

This study focused on optimizing the application of VCap in the diagnosis of Chronic Obstructive Pulmonary Disease, aiming to overcome the limitations of traditional pulmonary function tests, especially for vulnerable populations such as the elderly, children, and patients with severe respiratory distress. The primary scientific problem is to assess the effectiveness and reliability of VCap in COPD assessment . We proposed converting VCap into two-dimensional images and embedding them into a deep learning framework. Firstly, a novel quantitative analysis method for capnography was proposed, where volumetric capnography sequences were transformed into two-dimensional images using the Gramian Angular Field (GAF). To enrich the dataset and enhance the model’s generalizability, two data augmentation strategies were implemented: gaussian noise addition and elastic transformation. Lastly, CapnoNet was designed with multi-scale convolutional layers to interpret the complex information encoded in the transformed volumetric capnography images. In our findings, CapnoNet achieved an F1 score of 95.45% for COPD detection. For the task of COPD severity grading, the model demonstrated an F1 score of 89.15%.

This work presented a novel approach for the detection and severity grading of COPD using transformed volumetric capnography. The contributions of this study are as follows:

(1) A novel quantitative analysis method was proposed, transforming volumetric capnography into two-dimensional grayscale images using the Gramian Angular Field (GAF) transformation.
(2) CapnoNet, a multi-scale convolutional neural network, was developed to classify and grade the severity of COPD from the transformed capnograms.
(3) Data augmentation techniques, including the incorporation of Gaussian noise and elastic transformation, were employed to enhance the model’s robustness and generalizability across diverse clinical scenarios.
(4) The proposed approach demonstrated a diagnostic accuracy of 95.83% in detecting COPD and an accuracy of 96.36% in grading the severity of the disease.

The structure of this manuscript is organized as follows: Section 2 describes the methodology, including data collection, preprocessing, and the development of our convolutional neural network, CapnoNet. Section 3 presents the results of our model’s performance in detecting and grading COPD. Section 4 discusses the implications of these findings, potential limitations, and future research directions. Finally, Section 5 concludes with a summary of our contributions and their significance to the field of respiratory diagnostics.

## 2. Methods

### 2.1. Data Acquisition

In previous work [23], we designed and implemented a portable sensing device to accurately collect volumetric capnography data. This device primarily consists of a differential pressure sensor and a carbon dioxide concentration sensor based on non-dispersive infrared (NDIR) technology. To advance the device’s capabilities, we incorporated a Bluetooth communication module, enabling wireless data transmission. Additionally, we developed dedicated client software to facilitate efficient signal collection and management, providing a seamless user experience. The chosen differential pressure sensor, SDP32 from Sensirion (Stäfa, Switzerland), offers a measurement range of ±125 Pa. This range is optimal for recording the gas flow rate in real-time, capturing subtle pressure changes as the exhaled gas passes through a throttling device, which is critical for accurate volumetric analysis. The carbon dioxide concentration sensor (SprintIR^®^-W, Shenzhen, China) monitors and analyzes concentration changes in carbon dioxide non-invasively during respiration. The NDIR sensor operates by exploiting the unique absorption characteristics of carbon dioxide molecules at specific infrared wavelengths. This method is highly effective for carbon dioxide sensing because it allows for precise quantification based on the reduction in light intensity, directly correlating with the gas’s concentration. Prior to data collection, we undertake rigorous preparation steps, including a thorough check of the device’s airway integrity to prevent any sampling errors. Additionally, we calibrate the sensors to ensure their accuracy and reliability throughout the data collection process. Strict guidelines are established for patients during the data acquisition process. These guidelines instruct patients to avoid actions like coughing or obstructing the mouth, which could lead to incomplete or abnormal waveforms.

### 2.2. Data Preprocessing

**Figure 1:**
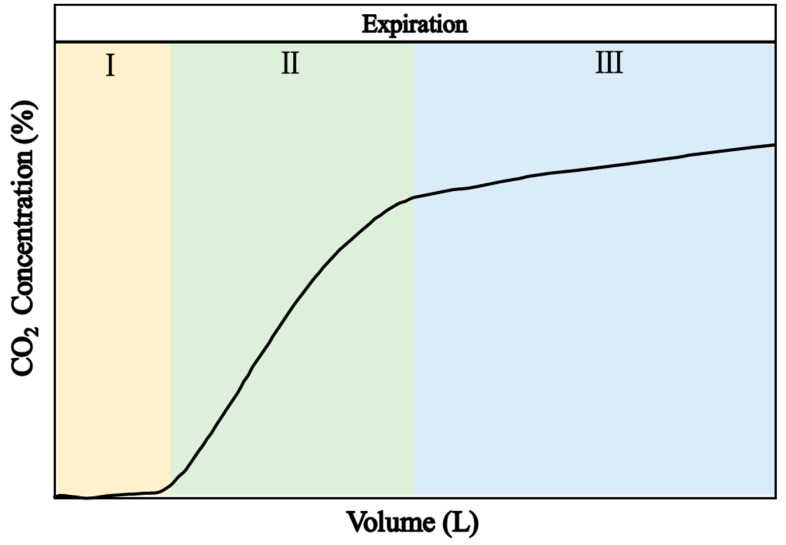
The waveform of volumetric capnography.

The volumetric capnogram is divided into three essential phases, as shown in Figure 1. Phase I is the baseline, representing the exhalation of gas from anatomical dead space that contains minimal to no carbon dioxide; Phase II is the ascending branch, characterized by the mixing and exhalation of gas from both dead space and alveoli, resulting in an increasing carbon dioxide concentration; Phase III is the plateau, representing alveolar gas with a high concentration of CO_2_. This phase is nearly horizontal in individuals without respiratory pathology, although its slope can reveal valuable clinical information.

**Figure 2:**
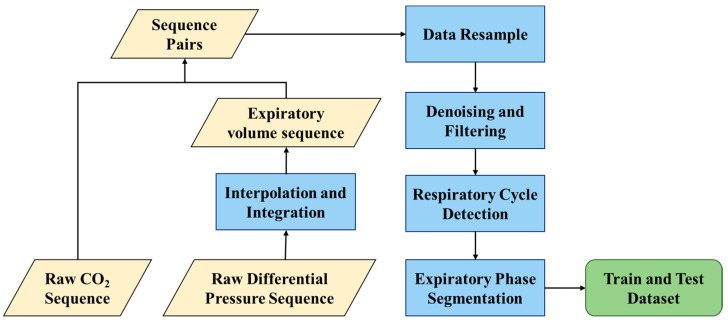
The diagram of data preprocessing.

To obtain a complete VCap waveform, the raw signals were preprocessed. Figure 2 demonstrates the signal preprocessing procedure. The raw signals, collected by the device, include sequences of carbon dioxide concentration and flow rate. Through interpolation, these sequences are temporally aligned, allowing for the integration of CO_2_ concentration data with corresponding flow rates to produce paired values of carbon dioxide concentration and exhaled volume. Subsequently, the data are resampled at 200 Hz, a rate chosen to balance detail and processing efficiency. A third-order Butterworth low-pass filter is then applied to diminish power frequency interference, a common type of noise in electronic signal collection, while retaining the essential features of the respiratory signal. To acquire standardized VCap waveforms, long-term respiratory signals are segmented. After identifying a natural and calm respiratory cycle, the peaks and troughs of the signal are detected, and the exhalation phase signal is extracted as a valid record. The segmented VCap curves are subsequently simplified through Piecewise Aggregate Approximation (PAA), a technique that condenses long sequences into shorter, representative segments. This process involves standardizing the length of the record to 224 samples, a strategy designed to maintain the original data trends and minimize the loss of critical diagnostic information. 

### 2.3. Gramian Angular Visualization of Volumetric Capnography

The Gramian Angular Field (GAF) represents time series data as a two-dimensional matrix, offering an image-based method [24]. In this study, we transformed volumetric capnography waveforms into grayscale images by GAF to improve the detection of respiratory abnormalities and aiding in clinical diagnostics.

Assuming the preprocessed sequence of volumetric capnography is represented as $X=\left(x_{1},x_{2},\ldots ,x_{n}\right)$, where n is the number of data samples, the initial sequence is normalized to [−1, 1], which is usually achieved through the following formula:

(1) $$\tilde{x_{i}}=\frac{2\left(x_{i}-\min\left(X\right)\right)}{\max\left(X\right)-\min\left(X\right)}-1$$

$\tilde{x_{i}}$ is the normalized signal sequence, *x_i_* is the original sequence.

Subsequently, each data point is converted to polar coordinates with the inverse cosine function, where $\theta_{i}$ denotes the angle and $r_{i}$ represents the radius.

(2) $$\phi_{i}=\arccos\tilde{x_{i}}$$

(3) $$r_{i}=\frac{t_{i}}{N}$$

This conversion to polar coordinates encodes the carbon dioxide volume sequence without loss. Among them, $\theta_{i}$ preserves numerical relationships, and $r_{i}$ ensures temporal consistency.

GAF is categorized into two types based on distinct inner product calculations: the Gramian Angular Summation Field (GASF) and the Gramian Angular Difference Field (GADF).

GASF calculates the inner product as $\left\langle x_{i},x_{j}\right\rangle =cos\left(\phi_{i}+\phi_{j}\right)$, where the inner product of two data points is the cosine of the sum of their polar angles, in the form of:

(4) $$GASF=\left[\begin{array}{cccc} \cos\left(\phi_{1}+\phi_{1}\right) & \cos\left(\phi_{1}+\phi_{2}\right) & \ldots & \cos\left(\phi_{1}+\phi_{n}\right)\\ \cos\left(\phi_{2}+\phi_{1}\right) & \cos\left(\phi_{2}+\phi_{2}\right) & \ldots & \cos\left(\phi_{2}+\phi_{n}\right)\\ \ldots & \ldots & \ldots & \ldots \\ \cos\left(\phi_{n}+\phi_{1}\right) & \cos\left(\phi_{n}+\phi_{2}\right) & \ldots & \cos\left(\phi_{n}+\phi_{n}\right) \end{array}\right]$$

Conversely, GADF determines the inner product $\left\langle x_{i},x_{j}\right\rangle =sin\left(\phi_{i}-\phi_{j}\right)$, where each element is the sine of the difference between two angles, resulting in a two-dimensional matrix as follows:

(5) $$GADF=\left[\begin{array}{cccc} \sin\left(\phi_{1}-\phi_{1}\right) & \sin\left(\phi_{1}-\phi_{2}\right) & \ldots & \sin\left(\phi_{1}-\phi_{n}\right)\\ \sin\left(\phi_{2}-\phi_{1}\right) & \sin\left(\phi_{2}-\phi_{2}\right) & \ldots & \sin\left(\phi_{2}-\phi_{n}\right)\\ \ldots & \ldots & \ldots & \ldots \\ \sin\left(\phi_{n}-\phi_{1}\right) & \sin\left(\phi_{n}-\phi_{2}\right) & \ldots & \sin\left(\phi_{n}-\phi_{n}\right) \end{array}\right]$$

When the exhaled carbon dioxide concentration varies with exhaled gas volume, GAF preserves the correlation between data points and the trend of waveform changes.

(6) $$\begin{array}{c} GASF=\left[\cos\left(\phi_{i}+\phi_{j}\right)\right]\\ ={\tilde{X}}^{'}\cdot \tilde{X}-\sqrt{I-{\tilde{X}}^{'2}}\cdot \sqrt{I-{\tilde{X}}^{2}} \end{array}$$

(7) $$\begin{array}{c} GADF=\left[\sin\left(\phi_{i}-\phi_{j}\right)\right]\\ =\sqrt{I-{\tilde{X\prime }}^{2}}\cdot \tilde{X}-X^{-}\sqrt{I-{\tilde{X}}^{2}} \end{array}$$

**Figure 3:**
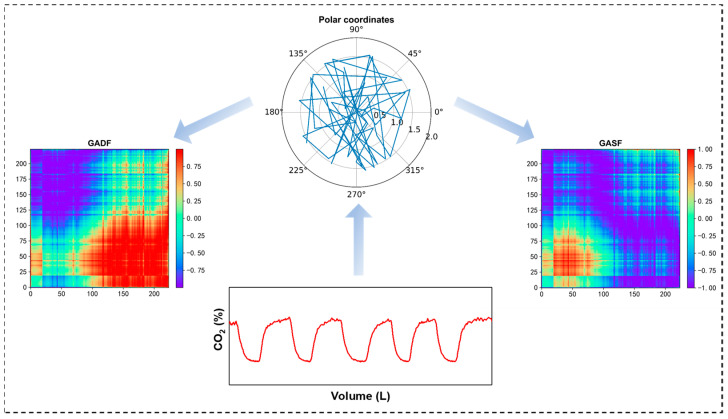
Gramian Angular Visualization of Volumetric Capnography.

Both the GASF and the GADF are bijective functions, meaning they establish a one-to-one correspondence between pairs of data points, ensuring that each point in one set is paired with exactly one point in another set, and vice versa. For clarity, the volumetric capnography transformed via the GASF and GADF methods will be referred to as GASF-VCap and GADF-VCap, respectively. As time progresses, the representations in the GASF-VCap and GADF-VCap matrices transition from the upper left to the lower right corner, effectively capturing and illustrating the temporal progression and dynamics of the original volumetric capnography signal within this two-dimensional space (as shown in Figure 3). This transformation not only elucidates specific inherent data characteristics, such as cyclic patterns and trends over time, but also aids in further modeling efforts by providing a structured, two-dimensional representation of complex temporal relationships.

### 2.4. Data Augmentation Strategy

**Figure 4:**
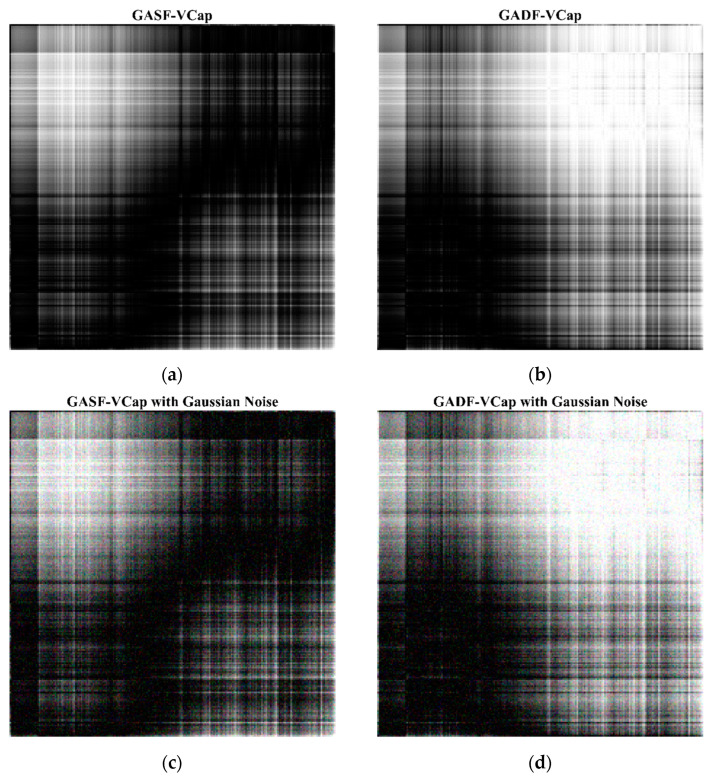

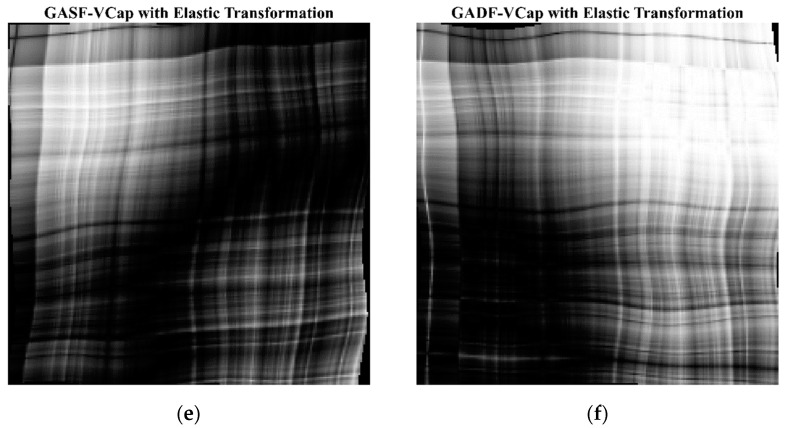
The results of data augmentation. (**a**) The volumetric capnography transformed via the GASF (GASF-VCap); (**b**) The volumetric capnography transformed via the GADF (GADF-VCap); (**c**) GASF-VCap with gaussian noise added; (**d**) GADF-VCap with gaussian noise added; (**e**) GASF-VCap with elastic transformation; (**f**) GADF-VCap with elastic transformation.

Data augmentation is an effective strategy for enhancing the performance of deep learning models by generating new data samples to increase the diversity of the dataset. In our study, two data augmentation strategies were employed: gaussian noise addition and elastic transformation. The addition of Gaussian noise is achieved by introducing random noise that follows a Gaussian distribution into the original data, with the mean and variance set to control the noise distribution. This method simulates the noise commonly encountered in actual data collection processes, thereby enhancing the model’s robustness to random perturbations. On the other hand, elastic distortion is used to mimic signal deformations in real-world scenarios. Elastic distortion involves adjusting parameters to control the degree of transformation. These include deformation intensity (which affects the magnitude of the distortions), displacement field smoothness (determining the spatial coherence of the distortions), and affine transformation intensity (adjusting the scale, rotation, and shear transformations). Through random and local non-linear transformations, elastic distortion simulates the possible morphological changes in capnography due to differences in respiratory patterns in the real world, thereby improving the model’s adaptability to shape variations. By combining these two methods, the training data were augmented to three times their original size, a factor determined through preliminary experiments to optimally balance between increasing dataset diversity and maintaining computational efficiency. Figure 4 displays the feature maps before and after applying data augmentation, showcasing how Gaussian noise and elastic distortion contribute to increased data variability. This visual comparison highlights the effectiveness of our data augmentation strategy in simulating real-world data variation.

### 2.5. Neural Network Architecture

**Figure 5:**
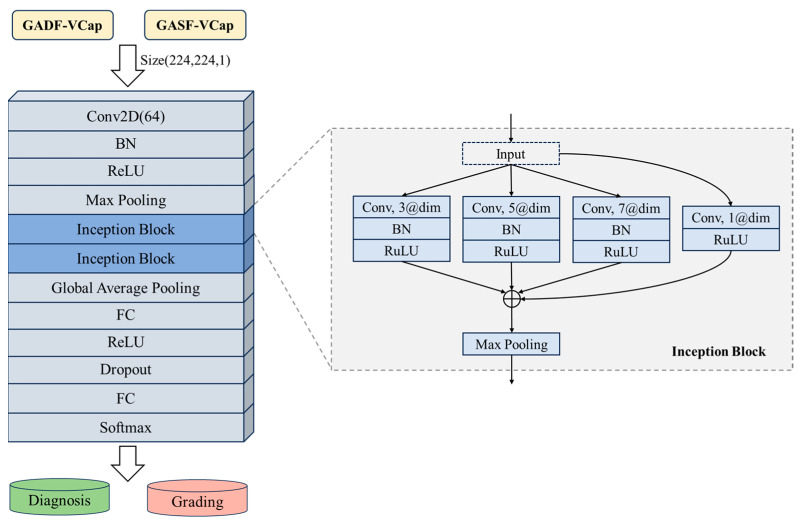
The architecture of CapnoNet.

The proposed model, CapnoNet, utilizes a Convolutional Neural Network (CNN) structure for the detection and grading of COPD. CapnoNet is designed with a compact, multi-scale convolutional backbone, centered on a foundational convolutional block and enhanced by the integration of two inception modules. Each inception module consists of four parallel processing branches, with the convolutional kernels sized at 1 × 1, 3 × 3, 5 × 5, and 7 × 7, respectively. In the architecture of the neural network, a Batch Normalization (BN) layer is incorporated subsequent to each convolutional layer. BN is a mechanism designed to enhance the stability and performance of neural networks. It achieves this by normalizing the inputs of each layer during the training process. This normalization helps to mitigate the internal covariate shift, thereby expediting the training process. This configuration allows for the extraction and refinement of features at multiple scales. To refine the feature representation, a 2 × 2 max pooling layer with a stride of 2 is integrated. This strategic design enables CapnoNet to effectively interpret capnography data by processing various kernel sizes simultaneously. The architecture is detailed in Figure 5, demonstrating the processing of 224 × 224 dimensional images. These images are generated from capnography signals transformed via the GAF method. The network begins with an initial 3 × 3 convolutional layer for preliminary feature extraction. This is followed by the application of two successive inception modules to deepen the feature analysis. Subsequently, a global average pooling layer prepares the extracted features for the final classification stage in the fully connected layer. To prevent overfitting, a dropout mechanism with a rate of 0.4 is applied after the fully connected layer. Additionally, all convolutional layers are designed with zero-padding to maintain the spatial dimensions of the input through each processing stage, ensuring that the network preserves the integrity of spatial resolution throughout its architecture.

### 2.6. Evaluation Metric

We use accuracy, precision, recall, and F1 score to evaluate the performance of models thoroughly. Accuracy indicates the rates of all samples are correctly classified. The formula is as follows:

(8) $$Accuracy=\frac{TP+TN}{TP+TN+FP+FN}$$

Recognizing the limitations of the accuracy metric in the context of unbalanced classes, we also evaluated our model using precision, recall, and the F1 score. These metrics are essential for a holistic assessment of model performance, especially in medical diagnostics where the cost of false negatives can be significant.

Precision indicates the rates of positive samples are correctly classified among positive predictions. The formula is as follows:

(9) $$Precision=\frac{TP}{TP+FP}$$

Recall indicates the rates of positive samples are correctly classified among samples that are truly positive. The formula is as follows:

(10) $$Recall=\frac{TP}{TP+FN}$$

where TP = true positive, which is the number of correctly classified positive samples; FP = false positive, incorrectly classified negative samples; TN = true negative, correctly classified negative samples, and FN = false negative, incorrectly classified positive samples.

Regarding the models’ composite performance, we used F1 scores for inter-indicator trade-offs and calculated them similarly for each task.

(11) $$F1\;score=\frac{2\times Precision\times Recall}{Precision+Recall}$$

## 3. Results

### 3.1. Subjects

In this study, a total of 1007 participants were enrolled. The assessments took place at the China-Japan Friendship Hospital, where participants underwent spirometry and volumetric capnography collection. Following the GOLD guidelines, a diagnosis of COPD was confirmed in individuals exhibiting a post-bronchodilator FEV1/FVC ratio of less than 0.7. The grading of airway obstruction was determined based on the ratio of actual to predicted FEV1 values, as detailed in Table 1.

Subjects demonstrating abnormal pulmonary functions underwent bronchodilator inhalation to assess the reversibility of airflow limitation, adhering strictly to clinical standards for Pulmonary Function Tests (PFTs). Records not meeting the established criteria were excluded from the study. Table 2 presents a descriptive analysis of the study participants, categorized into a total group (*n* = 1007), a normal group (*n* = 279), and a COPD group (*n* = 148). The COPD group was further divided into mild (*n* = 58), moderate (*n* = 51), severe (*n* = 34), and very severe (*n* = 5) subgroups based on their condition’s severity. During the capnography collection process, participants were instructed to breathe naturally and calmly. After identifying three natural breathing cycles, the formal signal collection commenced for a specified duration, repeated three times to ensure the consistency and reliability of the data collected.

### 3.2. Experimental Environment

The proposed model was developed using Python 3.11.7 and PyTorch 2.2.1, and all experimental validations were conducted on a system running Ubuntu 18.04, powered by an NVIDIA GeForce RTX 3090 GPU with 24 GB of RAM. 

### 3.3. Evaluation of the Proposed Model on COPD Detection

After preprocessing, the volumetric capnograms were transformed by GASF and GADF. These transformed data were then fed into CapnoNet for feature extraction, leveraging the network’s capability to identify significant patterns. The model employed the Adam optimizer to update network parameters and used the cross-entropy function as the loss function. Grid searching was utilized to find the optimal hyperparameters. The initial learning rate was set at 0.001, and the batch size at 32. Ten-fold cross-validation with stratified sampling was used to ensure that each fold of the dataset accurately represents the class distribution found in the entire dataset. 

The two-dimensional matrices obtained from GADF or GASF transformations (GADF-VCap or GASF-VCap) were used as inputs to CapnoNet, maintaining the same data preprocessing and model training procedures.

**Figure 6:**
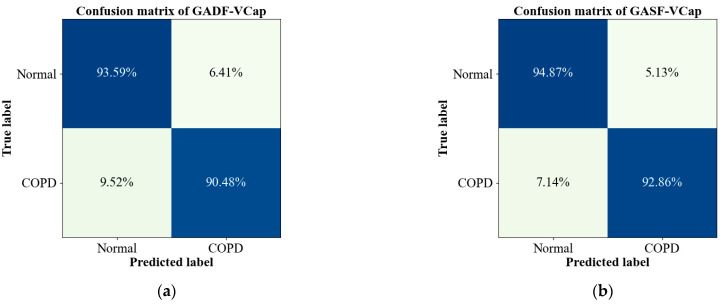
The confusion matrixes of the CapnoNet model on COPD detection. (**a**) The confusion matrix with GADF-VCap as model input; (**b**) The confusion matrix with GASF-VCap as model input.

Table 3 illustrates the performance on CapnoNet in detecting COPD. It is observed to effectively identify the COPD disease state and differentiate it from a healthy state. The model, when utilizing GASF-VCap as the input achieved its optimal performance, with an accuracy of 95.83%, a precision of 95.21%, a recall of 95.70%, and an F1 score of 95.45%. In the context of disease diagnosis, recall (or sensitivity) and the F1 score are of paramount importance as they indicate the model’s ability to correctly identify positive cases without missing those with the disease. Compared to using GADF-VCap as the input, it is observed that using GASF-VCap as the input improves the model’s performance in terms of recall and F1 score by 2.48% and 2.70%, respectively. This improvement is visually corroborated by the confusion matrix depicted in Figure 6, which illustrates GASF-VCap capability in recognizing both healthy subjects and those with COPD.

### 3.4. Evaluation of the Proposed Model on COPD Grading

**Figure 7:**
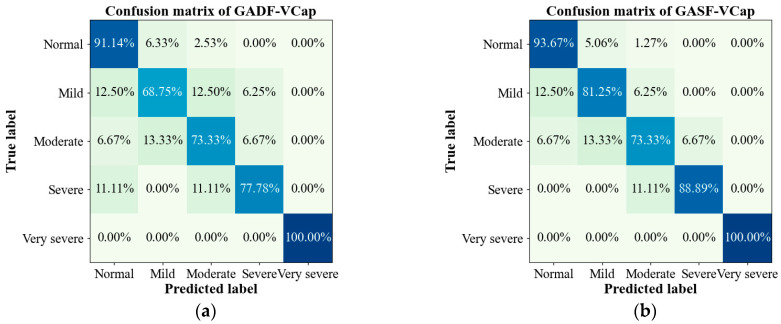
The confusion matrixes of the CapnoNet model on COPD grading. (**a**) The confusion matrix with GADF-VCap as model input; (**b**) The confusion matrix with GASF-VCap as model input.

Table 4 shows the results on COPD grading. The accuracy, precision, recall, and F1 score of the proposed model are 96.36%, 88.49%, 89.99%, and 89.15%, respectively. Figure 7 presents a confusion matrix to visually demonstrate the model’s accuracy across the different COPD severity levels. It is particularly noteworthy that CapnoNet exhibited good ability in identifying severe and very severe COPD patients. Additionally, when differentiating the more challenging mild COPD cases, CapnoNet managed to capture the subtle variations between different COPD stages. However, the model’s relatively lower performance in accurately identifying moderate COPD cases suggests a need for further refinement.

### 3.5. Evaluation of Data Augmentation Strategies

To assess the effectiveness of the proposed data augmentation strategies, GASF-VCap was employed as the source data input into the CapnoNet model for an ablation experiment. Gaussian noise was added to GASF-VCap, and elastic transformations were applied. Following data augmentation, the original training dataset was expanded to three times its size. Specifically, Gaussian noise with a mean of 0 and a variance of 15 was added to increase variability, and elastic transformations, which simulate natural variations in the data by stretching and distorting the input images, were applied with the parameters alpha set to 200 (indicating the intensity of the transformation), sigma at 20 (the elasticity coefficient), and alpha affine at 10 (controlling the affine transformations). The augmented feature maps are illustrated in Figure 4c,e, showcasing the effects of these parameters.

We compared the performance of the CapnoNet model on two critical tasks, both with and without the application of these data augmentation strategies. The tasks involved the detection and grading of COPD. The results, as detailed in Table 5, reveal a significant improvement in the model’s performance attributable to data augmentation. Specifically, the application of data augmentation resulted in an increase in the F1 scores by 1.83% and 2.37% for the respective tasks.

## 4. Discussion

In this study, we introduced a novel methodology for transforming VCap into a two-dimensional matrix to facilitate quantitative analysis. This approach utilizes the deep learning framework CapnoNet for the detection and grading of COPD. The results, as delineated in Table 3 and Table 4, substantiate the effectiveness of CapnoNet in accurately diagnosing COPD across varying degrees of complexity. A noteworthy aspect of our research is the employment of the GASF transformation strategy, which significantly enhanced CapnoNet’s diagnostic capabilities, achieving an impressive detection accuracy of 95.83%. Furthermore, our model demonstrated remarkable proficiency in distinguishing between different severities of COPD, thereby highlighting the substantial benefits of combining volumetric capnography visualization with deep learning analysis. However, our findings also illuminate a notable challenge (Figure 7b): the precise differentiation between mild and moderate stages of COPD remains arduous, revealing the nuanced complexities of ventilatory dysfunction in patients with COPD. This observation underscores the necessity for ongoing enhancements in model sensitivity to better discern these subtle distinctions.

By comparing the Gramian Angular Summation Field (GASF) and Gramian Angular Difference Field (GADF) transformation methods, we delved into the effects of different two-dimensional image representations on the performance of the models. The GASF-VCap model outperformed the GADF-VCap model across multiple performance metrics. This advantage is likely due to GASF’s retention of absolute position information in time series. Leveraging the cosine function’s properties, GASF effectively encapsulates the cumulative patterns present in time series data. In contrast, GADF, by computing sine values between points on the unit circle, reflects the rate of change within the time series. It is believed that GASF represents the overall trends and patterns in the capnography sequence, whereas GADF focuses more on the dynamic variations and relative positions in the capnograph sequence. Specific respiratory patterns in VCap, indicative of COPD and varying degrees of airway obstruction, are not only dependent on the depth and speed of breathing but are also closely related to the CO_2_ concentration at specific points in the respiratory cycle. By preserving the original structure of VCap series, GASF enables the model to capture these critical features, thereby enhancing diagnostic accuracy. The preservation of time series integrity and the detailed pattern information emerge as crucial for the effective diagnosis and grading of COPD. 

Furthermore, the evaluation of CapnoNet’s training and computational complexity, quantified by measuring its parameter size and floating-point operation (FLOP) requirements—comprising 1.02 million parameters and necessitating only 0.23 GFLOPs per forward pass—underscores the model’s potential for deployment in resource-constrained environments, enhancing its applicability in real-world scenarios. This aspect highlights the model’s suitability for widespread clinical use, especially in settings where computational resources are limited.

Nevertheless, this study has limitations. Firstly, our dataset was derived from a single center, lacking validation from out-of-hospital subjects, which may affect the external validity of the results. Secondly, the distribution of COPD patients of varying severity levels was uneven in our outpatient-based study population, particularly with fewer severe and very severe cases, limiting the model’s generalizability across a broader population. Moreover, although the CapnoNet network demonstrated potential in preliminary studies, as a relatively simplified deep learning architecture, it still has room for improvement and optimization. Given the potential of deep learning technology in handling complex medical data, future research will aim to expand the sample size, incorporate external validation sets, and further refine the network architecture to enhance the model’s accuracy and generalizability.

## 5. Conclusions

To the best of the authors’ knowledge, this study represents the first instance of volumetric capnography (VCap) data being analyzed through imaging techniques for quantitative analysis. A novel method was introduced for diagnosing and grading COPD using image-based capnography. By applying GAF transformation, capnography images are converted into two-dimensional grayscale images, and accurate COPD diagnosis and airway obstruction grading are achieved through a cascading CNN-Inception network. Furthermore, an image enhancement algorithm incorporating gaussian noise and elastic distortion is utilized to mitigate the variability of respiratory fluctuations, with ablation studies confirming the effectiveness of this strategy. The results on our own dataset demonstrate that the proposed CapnoNet model exhibits excellent classification performance, accurately distinguishing between COPD patients and healthy individuals and identifying different patterns of airway obstruction. In conclusion, this study is the first to transform traditional capnography data into image format, providing a new perspective for the quantitative analysis of VCap. The proposed CapnoNet model provides an effective tool for the early diagnosis and treatment of COPD. This work adeptly tackles the challenges that existed previously in COPD diagnosis. This advancement enables the enhanced remote monitoring and management of COPD patients, significantly contributing to improved healthcare delivery.

## Figures and Tables

**Table 1:** GOLD grades and severity of airflow obstruction in COPD (based on post-bronchodilator FEV1).

Grade	Severity	Airflow Obstruction
GOLD1	Mild	FEV1 ≥ 80% predicted
GOLD2	Moderate	50% ≤ FEV1 < 80% predicted
GOLD3	Severe	30% ≤ FEV1 < 50% predicted
GOLD4	Very Severe	FEV1 < 30% predicted

**Table 2:** Description of subjects.

	Total (*n* = 1007)	Normal (*n* = 279)	COPD (*n* = 148)	GOLD 1 (*n* = 58)	GOLD 2 (*n* = 51)	GOLD 3 (*n* = 34)	GOLD 4 (*n* = 5)
Male, n	535	132	102	40	35	22	5
Female, n	472	147	46	18	16	12	0
Age, mean ± SD	48 ± 38	51 ± 13	58 ± 10	58 ± 11	61 ± 10	56 ± 11	61 ± 10
Height, cm, mean ± SD	165.45 ± 8.58	166.00 ± 8.12	166.27 ± 9.20	168.24 ± 10.73	166.10 ± 7.84	162.71 ± 7.86	168.50 ± 5.01
Weight, kg, mean ± SD	68.58 ± 14.00	70.92 ± 16.60	68.71 ± 12.32	73.05 ± 12.21	68.02 ± 10.76	64.29 ± 12.63	56.83 ± 7.36
FEV1, L	2.52 ± 0.86	3.11 ± 0.67	1.85 ± 0.87	2.61 ± 0.75	1.67 ± 0.41	1.00 ± 0.26	3.65 ± 0.17
FVC, L	3.48 ± 1.00	3.85 ± 0.85	3.33 ± 1.16	4.16 ± 1.17	3.02 ± 0.80	2.55 ± 0.68	2.35 ± 0.34
FEV1/FVC, %	71.91 ± 12.70	81.05 ± 4.40	53.98 ± 12.18	62.86 ± 5.37	56.00 ± 7.12	40.26 ± 8.27	27.36 ± 4.95

FEV1 (forced expiratory volumn in one second): Volume that has been exhaled at the end of the first second of forced expiration. FVC (forced vital capacity): the determination of the vital capacity from a maximally forced expiratory effort.

**Table 3:** Performance of the proposed model in COPD detection.

Model Input	Accuracy	Precision	Recall	F1 Score
GADF-VCap	0.9333	0.9234	0.9322	0.9275
GASF-VCap	0.9583	0.9521	0.9570	0.9545

**Table 4:** Performance of the proposed model in COPD grading.

Model Input	Accuracy	Precision	Recall	F1 Score
GADF-VCap	0.9471	0.8375	0.8567	0.8459
GASF-VCap	0.9636	0.8849	0.8999	0.8915

**Table 5:** Data Augmentation results on GASF-VCap.

Task	DA	Accuracy	Precision	Recall	F1 Score
COPD Detection	no	0.9417	0.9340	0.9386	0.9362
	yes	0.9583	0.9521	0.9570	0.9545
COPD Grading	no	0.9570	0.8640	0.8743	0.8678
	yes	0.9636	0.8849	0.8999	0.8915

DA: data augmentation strategies.

## Data Availability

The data that support the findings of this study are available from the corresponding author and the first author upon reasonable request.
